# Molecular Surveillance for Multidrug-Resistant *Plasmodium falciparum*, Cambodia

**DOI:** 10.3201/eid1410.080080

**Published:** 2008-10

**Authors:** Naman K. Shah, Alisa P. Alker, Rithy Sem, Agustina Ika Susanti, Sinuon Muth, Jason D. Maguire, Socheat Duong, Frederic Ariey, Steven R. Meshnick, Chansuda Wongsrichanalai

**Affiliations:** University of North Carolina School of Public Health, Chapel Hill, North Carolina, USA (N.K. Shah, A.P. Alker, S.R. Meshnick); National Center for Parasitology, Entomology and Malaria Control, Phnom Penh, Cambodia (R. Sem, S. Muth, S. Duong); Pasteur Institute of Cambodia, Phnom Penh, (R. Sem, F. Ariey); US Naval Medical Research Unit No. 2, Jakarta, Indonesia (A.I. Susanti, J.D. Maguire, C. Wongsrichanalai)

**Keywords:** plasmodium falciparum, antimalarial drugs, drug resistance, Cambodia, genetic markers, pfmdr1 protein, surveillance, artesunate, mefloquine, malaria, dispatch

## Abstract

We conducted surveillance for multidrug-resistant *Plasmodium falciparum* in Cambodia during 2004–2006 by assessing molecular changes in *pfmdr1*. The high prevalence of isolates with multiple *pfmdr1* copies found in western Cambodia near the Thai border, where artesunate–mefloquine therapy failures occur, contrasts with isolates from eastern Cambodia, where this combination therapy remains highly effective.

The Thailand–Cambodia border has been an epicenter for drug-resistant *Plasmodium falciparum*. Recent clinical studies indicate that efficacy of artesunate–mefloquine combination is decreasing on both sides of the border (*1*–*3*). In contrast, *P. falciparum* in eastern Cambodia remains sensitive to mefloquine and the artesunate–mefloquine combination (*4*,*5*). Declining artesunate–mefloquine efficacy on the Thai border and the geographic variation in susceptibility to this combination therapy suggest expanded surveillance is needed in Cambodia. An inexpensive method to help target in vivo monitoring sites is surveillance for molecular markers of drug resistance.

We have previously shown that elevated *P. falciparum* multidrug resistance 1 (*pfmdr1*) gene copy number is associated with an 8-fold risk for artesunate–mefloquine failure in western Cambodia (*6*). More recently, we conducted a molecular surveillance for *pfmdr1* mutations to guide the selection of sentinel sites for in vivo monitoring of artesunate–mefloquine resistance.

## The Study

**Figure 1:**
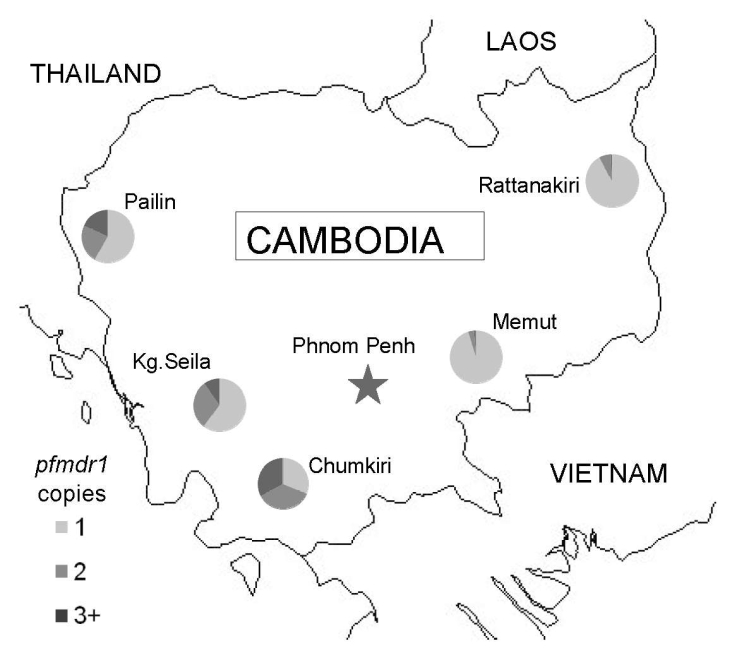
Map of Cambodia with locations of surveillance sites and proportion of isolates containing 1, 2, or >3 copies of *pfmdr1*, May 2004–December 2006.

Clinical isolates of *P. falciparum* were collected from 5 sites across Cambodia (Figure 1): Pailin, Kampong Seila, Chumkiri (western), Memut, and Rattanakiri (eastern) during 2004–2006. Study participants included patients who were seen at health centers with uncomplicated falciparum malaria, including mixed infections. Fifty-six samples from patients in Pailin had been previously analyzed (*6*). Institutional review board approvals were obtained from the Cambodian National Ethics Committee for Health Research, the US Naval Medical Research Unit No. 2, and the University of North Carolina at Chapel Hill (UNC).

Peripheral blood was used to prepare smears and filter paper blood spots (25 μL, 2 spots) on Whatman 3-mm blotting paper (Whatman International Ltd., Maidstone, Kent, UK) from each participant. Blood smears were examined to determine parasite density, nonfalciparum species, and gametocytes. Enrolled participants completed a questionnaire on demographic information and past medical history. They received artesunate–mefloquine in accordance with national guidelines for uncomplicated falciparum malaria. DNA was extracted from blood spots by using QIAmp Mini kits (QIAGEN, Valencia, CA, USA). *pfmdr1* copy number and single-nucleotide polymorphism assays were performed as previously described (*6*,*7*), except that in *pfmdr1* single-nucleotide polymorphism assays the concentration of probes was reduced from 250 nM to 125 nM and reactions were not duplicated.

Linear regression was used to determine if *pfmdr1* copy number varied by site. *pfmdr1* copy number was inverse transformed to meet assumptions of normality and homoscedasticity. Backwards elimination based on a p value of 0.05 was used to determine the effect of site when controlling for confounders. Logistical regression was used to confirm the results of the linear regression analysis by using a cutoff of 1.5 copies (*8*). Logistic regression was also used to determine if *pfmdr1*-184 genotype varied by site. Samples with a mixture of 184-phe and 184-tyr were coded as 184-phe. All statistical analyses were conducted in Stata 8.2 (StataCorp, College Station, TX, USA).

We enrolled 744 study participants with uncomplicated *P. falciparum* malaria. The characteristics of participants at each site are described in Table 1. *pfmdr1* copy number was successfully determined for 712 (95.7%) of 744 samples. When compared with results at UNC, results of copy number assays performed by the National Malaria Control Program (NMCP) staff in Phnom Penh were slightly lower (mean difference –0.163, 95% confidence interval [CI] –0.277 to –0.049, n = 44), and the 2 groups were in 100% concordance with distinguishing samples with >2 *pfmdr1* copies.

**Table 1:** Clinical characteristics of study participants at 5 surveillance sites, Cambodia, May 2004–December 2006*

Characteristic	Pailin	Kg. Seila†	Chumkiri	Memut	Rattanakiri
Total no.	146	11	116	172	299
Male, no. (%)	98 (67.1)	9 (81.8)	97 (83.6)	62 (36.1)	187 (62.5)
Adult (>18 y), no. (%)	128 (87.7)	11 (100.0)	109 (94.0)	172 (100.0)	221 (73.9)
Parasitemia,‡ geometric mean	15,001	5,975	13,503	19,800	22,443
Parasitemia >50,000, no. (%)	47 (32.2)	4 (36.4)	33 (28.7)	68 (39.8)	120 (40.4)
Gametocytemia, no. (%)	8 (5.5)	1 (9.0)	11 (9.6)	12 (7.0)	8 (2.7)
Smear-positive malaria in past y, no. (%)	35 (39.3)	2 (20.0)	25 (61.0)	13 (7.6)	127 (46.7)
Antimalarial drug taken in past mo, no. (%)	13 (14.4)	2 (18.2)	20 (17.2)	64 (37.2)	6 (2.0)
Mixed infection, no. (%)	6 (4.1)	1 (9.1)	8 (7.0)	3 (1.8)	6 (2.0)
No. d of illness, median (range)	3 (1–6)	4 (2–6)	6 (1–6)	4 (2–6)	4 (0–6)

*The missing values by category include parasitemia (n = 4), gametocytemia (n = 4), smear-positive malaria in past year (n = 160), antimalarial drug taken in past month (n = 56), mixed infection (n = 4), and no. of d of illness (n = 21). †Baseline differences were most likely due to small sample size. ‡Parasites/μL.

**Figure 2:**
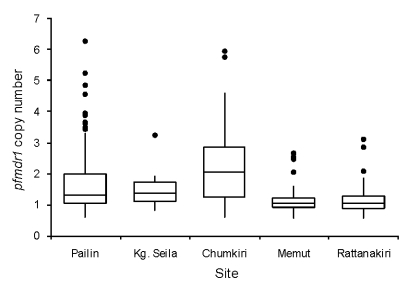
Median values and interquartile ranges of *pfmdr1* copy number across 5 surveillance sites in Cambodia, May 2004–December 2006. Kg., Kampong.

*pfmdr1* copy numbers ranged from 0.6 to 6.3, and 167 (23.5%) of 712 samples had >1.5 copies. The median and distribution of *pfmdr1* copy number varied by site and were highest in the 3 western Cambodian sites (Figure 2). The proportion of samples with >1.5 *pfmdr1* copies was greater in western than eastern Cambodia (52.7% vs. 6.4%, odds ratio [OR] = 16.2, 95% CI 10.3–25.3). The prevalence of parasite samples with amplified *pfmdr1* varied by site and differed in the prevalence of 2 and >3 copies of *pfmdr1* (Figure 1).

The prevalence of *pfmdr1*-184-Phe varied by site, was significantly higher in the West (85.5% vs. 17.1%, OR = 27.4, 95% CI 18.1–41.4), but was not more prevalent in samples with *pfmdr1* copy number >1.5 when site was controlled for (OR = 1.5, 95% CI 0.9–28, p = 0.139). *Pfmdr1* codons 86, 1034, and 1042 were predominantly wild-type, 356 (98.6%) of 361, 215 (97.7%) of 220, 347 (95.9%) of 362, respectively, with little variation between sites (data not shown). Most (196 [91.2%] of 215) samples had the haplotypes 86-Asn, 1034-Ser, and 1042-Asn.

In univariate and multivariate linear regression models, site was the strongest predictor of *pfmdr1* copy number. Chumkiri had the highest *pfmdr1* copy number before and after clinical correlates were controlled for (Table 2). The relationship between copy number and site was retained when the analysis was repeated with logistic regression. Compared with Rattanakiri, the OR of having a copy number >1.5 was 32.4 (95% CI 16.4–64.2) for Chumkiri, 7.8 (95% CI 4.3–13.9) for Pailin, and 0.5 (95% CI 0.2–1.2) for Memut when parasitemia and year were controlled for.

**Table 2:** Univariate and multivariate linear regression analyses of the association between surveillance site and *pfmdr1* copy no., Cambodia

Site	No.	Mean	Univariate linear regression			Multivariate linear regression*		
			β†	95% CI‡		Predicted mean	β†	95% CI‡
Rattanakiri	285	1.09	0			1.03	0	
Memut	165	1.09	–0.001	–0.056 to 0.053		1.02	0.047	–0.019 to 0.112
Kampong Seila	10	1.53	–0.234	–0.412 to –0.055		1.35	–0.184	–0.363 to –0.006
Pailin	143	1.70	–0.232	–0.289 to–0.175		1.34	–0.217	–0.276 to –0.159
Chumkiri	109	2.24	–0.393	–0.456 to –0.331		1.72	–0.432	–0.503 to –0.362

*Controlled for parasitemia and year. †The coefficient estimating the relationship between site and inverse-transformed *pfmdr1* copy no. ‡CI, confidence interval.

## Conclusions

We found geographic heterogeneity for 2 *pfmdr1* genetic markers: *pfmdr1* 184 and *pfmdr1* amplification. Genotype 184-Phe was associated with *pfmdr1* amplification at the population level but not in individual isolates. The basis of this trend is unknown. Pailin, Kampong Seila, and Chumkiri in western Cambodia had higher *pfmdr1* copy numbers and higher prevalence of *pfmdr1* 184-Phe than Memut and Rattanakiri in eastern Cambodia. An association between these markers and resistance is consistent with reports of reduced efficacy of artesunate–mefloquine in Pailin (79% over 42 days) (*2*) compared with Memut and Rattanakiri, where artesunate–mefloquine efficacy remains close to 100% (*4*,*5*). This finding is also consistent with the decrease of mefloquine sensitivity in western Cambodia based on in vitro drug susceptibility monitoring conducted by the Pasteur Institute of Cambodia from 2001 through 2007 (*9**,**10*; P. Lim, pers. comm.).

Chumkiri was surveyed because local health staff observed that falciparum malaria patients treated with artesunate–mefloquine often returned within weeks with recurrent fever and parasitemia. The possibility of such resistance in this site was particularly alarming because it was thought to be confined to Thailand–Cambodia border areas. Chumkiri had not previously been a sentinel site for antimalarial efficacy monitoring by NMCP. On the basis of these *pfmdr1* data, a clinical trial of artesunate–mefloquine was launched in Chumkiri in 2006 (*11*). Clinical validation of predicted resistance by in vivo studies is beyond the scope of this study, although multiple in vitro and in vivo studies have consistently shown an association of increased *pfmdr1* copy number with mefloquine or artesunate–mefloquine failure in Southeast Asia (*8*,*10*,*12*–*15*), supporting use of *pfmdr1* copy number in routine surveillance and policy formation.

Monitoring changes in antimalarial drug efficacies is essential for guiding treatment policies in an era of multidrug resistance. However, such studies are resource intensive. In Cambodia, for example, NMCP can conduct in vivo studies at only 2–3 sites per year because of limited funds and trained staff. Molecular markers can help target in vivo studies where they are most needed. Molecular surveillance is high-throughput and can be performed in a central laboratory on dried blood spots. Expanded surveillance allows for molecular mapping and enables a rapid containment of resistance foci. *pfmdr1* assays are now routinely performed in Cambodia by NMCP staff. National and regional molecular surveillance by malaria-endemic countries is a real possibility.
